# The Role of Mre Factors and Cell Division in Peptidoglycan Growth in the Multicellular Cyanobacterium *Anabaena*

**DOI:** 10.1128/mbio.01165-22

**Published:** 2022-07-25

**Authors:** Cristina Velázquez-Suárez, Ana Valladares, Ignacio Luque, Antonia Herrero

**Affiliations:** a Instituto de Bioquímica Vegetal y Fotosíntesis, CSIC and Universidad de Seville, Seville, Spain; University of Nebraska-Lincoln

**Keywords:** cell growth, *Anabaena* filaments, intercellular septa, lateral peptidoglycan growth, septal peptidoglycan growth

## Abstract

Bacteria in general serve two main tasks: cell growth and division. Both processes include peptidoglycan extension to allow cell expansion and to form the poles of the daughter cells, respectively. The cyanobacterium *Anabaena* forms filaments of communicated cells in which the outer membrane and the peptidoglycan sacculus, which is engrossed in the intercellular regions between contiguous cells, are continuous along the filament. During the growth of *Anabaena*, peptidoglycan incorporation was weak at the cell periphery. During cell division, midcell peptidoglycan incorporation matched the localization of the divisome, and incorporation persisted in the intercellular septa, even after the division was completed. MreB, MreC, and MreD were located throughout the cell periphery and, in contrast to other bacteria, also to the divisome all along midcell peptidoglycan growth. In *Anabaena* mutants bearing inactivated *mreB, mreC*, or *mreD* genes, which showed conspicuous alterations in the filament morphology, consecutive septal bands of peptidoglycan growth were frequently not parallel to each other and were irregularly spaced along the filament, reproducing the disposition of the Z-ring. Both lateral and septal growth was impaired in strains down-expressing Z-ring components, and MreB and MreD appeared to directly interact with some divisome components. We propose that, in *Anabaena*, association with the divisome is a way for localization of MreB, MreC, and MreD at the cell poles, where they regulate lateral, midcell, and septal peptidoglycan growth with the latter being involved in localization and maintenance of the intercellular septal-junction protein structures that mediate cell-cell communication along the filament.

## INTRODUCTION

Bacterial activity ultimately serves two main tasks: cell growth and division. Bacterial cells are encircled by distinct envelopes that compartmentalize the intracellular milieu and protect it from external insults while also permitting exchanges with the surrounding environment. Both cell growth and cell division processes must include an extension of the cell envelope to allow cell enlargement and daughter cell separation, respectively. A component of the bacterial cell envelope is the murein, an elastic sacculus that surrounds the cell outside the cytoplasmic membrane, allowing it to cope with osmotic challenges and playing a pivotal role in the determination of the strain-specific morphology.

The murein is a giant peptidoglycan (PG) molecule made of long glycan strands of alternating *N*-acetylglucosamine and *N*-acetylmuramic acid units cross-linked by short peptides. It occurs as a single monolayer in common Gram-negative bacteria, in which the PG is placed in the periplasm between the cytoplasmic and outer membranes and is multilayered in Gram-positive bacteria. The synthesis of PG involves cytoplasmic and inner membrane-linked steps to form the lipid-anchored disaccharide pentapeptide (lipid II), the building block for PG synthesis, which is thereafter flipped to the outer leaflet of the plasma membrane. Outside of the cell (in the periplasm in Gram-negative bacteria), the lipid II precursor is polymerized into a PG growing strand by membrane-bound glycosyl transferases (which catalyze the bonding of precursor units to the glycosidic backbone with the release of the lipid moiety) and transpeptidases that cross-link the peptide units in adjacent PG strands (1, 2).

The spatiotemporal pattern of PG synthesis is tightly regulated according to the growth cycle and the environment, and this can be achieved by the organization of PG-processing enzymes in multiprotein complexes that are directed by cytoplasmic cytoskeletal proteins to specific subcellular localizations. Thus, at the initiation of cell division, the tubulin structural homolog FtsZ is bound to the inner membrane by interaction with diverse membrane-associated proteins and polymerizes to form a dynamic ring of short filaments that contract and move circumferentially at the future site of division by a head-to-tail treadmilling mechanism that drives the movement of the PG-processing machinery directing midcell PG incorporation to form the new poles of the daughter cells (2–5). In addition, in most rod-shaped bacteria, the actin structural homolog MreB initiates the assembly of the elongasome, which directs a dispersed incorporation of PG in the cylindrical part of the cell wall allowing the growth of newly divided cells (2, 6).

The subcellular organization and localization of the MreB macromolecular complexes have been debated, being proposed to form cell-spanning helical structures or discrete patches of filaments with variable lengths (see reference (7)). In any case, the movement of MreB filaments, together with the integral membrane protein MreD and the periplasmic MreC, which also forms patches or short filaments (MreC from Pseudomonas aeruginosa has been recently shown to form tubular filaments *in vitro*; (7)), would guide the spatial localization of both the cytoplasmic enzymatic complex for PG precursor synthesis and the periplasmic PG-processing enzymes to insert new PG circumferentially, which in turn would contribute to the motion of MreB filaments, resulting in cell elongation (e.g., (8–11)).

In both Escherichia coli (12) and Caulobacter crescentus (13), in addition to septal and lateral growth, a midcell-localized PG synthesis takes place at the initiation of cell division leading to ring-like cell elongation at prospective division sites. In both bacteria, preseptal PG growth requires FtsZ, and, in E. coli, ZipA (a secondary FtsZ membrane tether) (13, 14). Mre proteins have been observed to form rings adjacent to the FtsZ ring at the initiation of cell division (15–21).

In addition, studies on other bacteria with different cell morphologies, such as the spherical Staphylococcus aureus (22) and the ovococcus Streptococcus pneumoniae, (23, 24), or even the rod-shaped Corynebacterium glutamicum (25), have revealed an ample diversity in the spatial and temporal pattern of localization of PG growth.

Filamentous heterocyst-forming cyanobacteria, such as those in the *Anabaena* genus, represent paradigmatic cases of bacterial multicellularity, in which the organismic unit was shifted from the individual cells to the filament (26, 27). The *Anabaena* filament is composed of tens to hundreds of cylindrical cells, which may include different cell types, that communicate by two paths allowing intercellular molecular exchange throughout the filament: a shared periplasm, which is established by continuity of the outer membrane, and proteinaceous septal channels that traverse the cell envelope at the septa of contiguous cells (26). In *Anabaena*, the PG sacculus appears to be continuous along the filament, surrounding each cell and is engrossed at the intercellular septa between the cytoplasmic membranes of the daughter cells resulting from intercalary cell division (28). Indeed, PG sacculi fragments encompassing several cells can be readily isolated (e.g., (28)). Notably, the PG intercellular septal caps are perforated by nanopores, which have been proposed to hold the septal junction proteins that communicate the contiguous cells (28, 29). Despite the distinct structural features of *Anabaena*, relatively little is known about the mechanisms of cell elongation and cell division that operate to produce adjoined and communicated cells in comparison to the available detailed knowledge of these processes in unicellular bacteria.

Regarding elongasome components, *Anabaena* possesses homologs to MreB, MreC, and MreD factors, and mutants with inactivated *mre* genes present cells bigger than those of the wild-type and, especially in the case of the *mreC* mutant, stretches of filaments with cells of different sizes, indicating a role of MreB, MreC, and MreD in cell size control (30). The cells of the three mutants are more rounded than those of the wild-type and, contrary to the latter, in the mutant cells, the axis parallel to the filament is shorter than the axis perpendicular to the filament (30, 31). Cell enlargement and inversion of cell axes in the filament were also reported for an *Anabaena* derivative with inactivated ORF alr5045 encoding a PG transpeptidase, thus suggesting its involvement in cell elongation (32).

Regarding cell division proteins, *Anabaena* presents homologs of some of the widespread bacterial divisome components, together with specific cyanobacterial components. As in most other bacteria, cell division initiates by the polymerization of a Z-ring. However, *Anabaena* FtsZ has an N-terminal peptide specific to filamentous heterocyst-forming strains that is essential for cell division and appears to determine a distinct FtsZ polymerization mode (33). On the other hand, the cyanobacterium-specific protein ZipN represents the main FtsZ tether to the cytoplasmic membrane and the main organizer of the *Anabaena* divisome (34). Otherwise, little has been investigated on further divisome functions, except the recruitment of septal proteins involved in intercellular communication by specific interactions with divisome components during cell division (34–36), and the involvement of AmiC amidases in building the septal PG nanopore arrays (37–39).

In this work, we aimed at getting advance on the knowledge of the modes of PG growth operating in *Anabaena*, including the influence on these processes of the MreB, MreC, MreD, FtsZ, and ZipN proteins, and their involvement in cell growth and the determination of the cell morphology and the structure of the filament.

## RESULTS

### Topology of PG growth in *Anabaena*.

**FIG 1:**
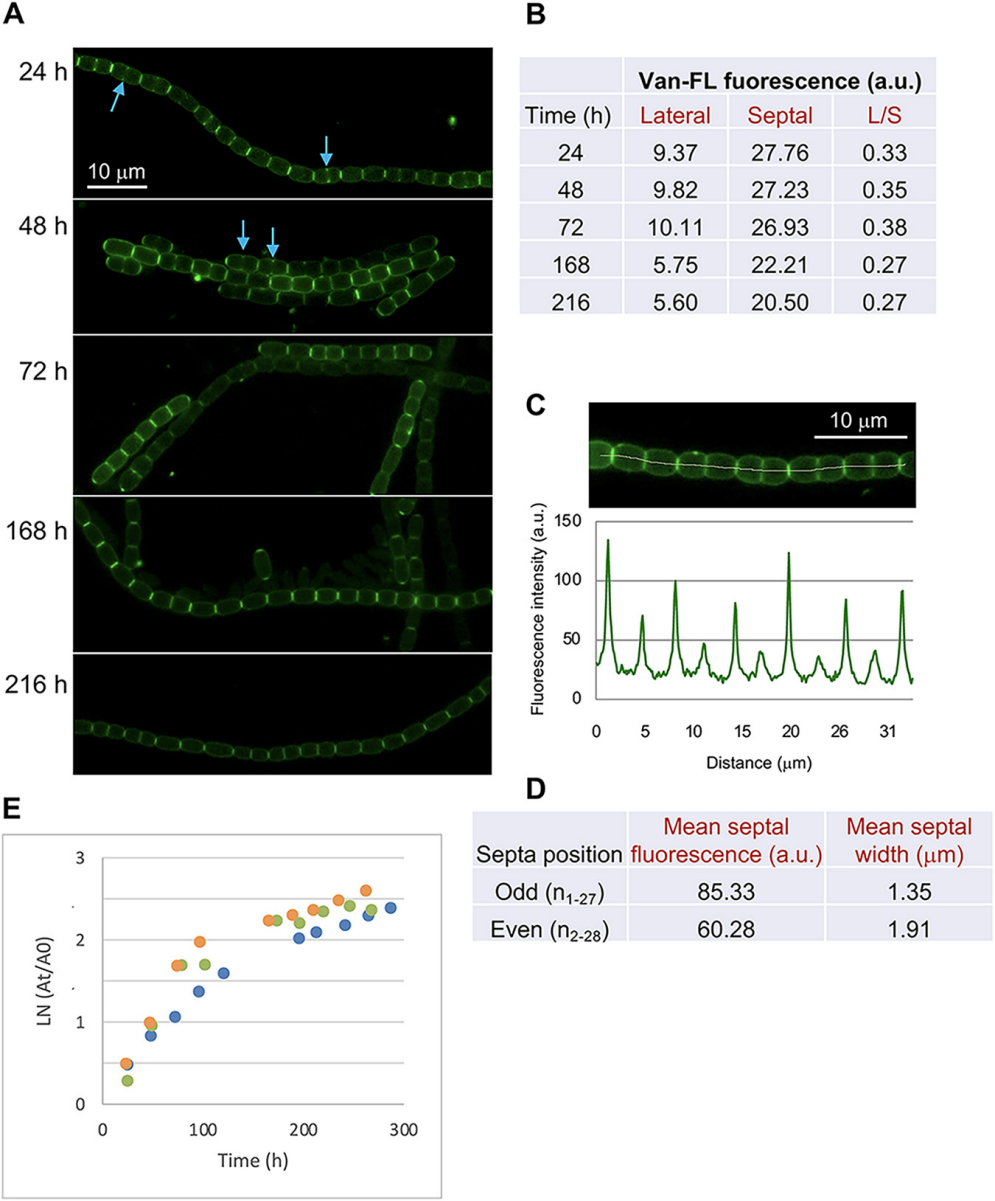
Van-FL staining of *Anabaena*. Exponential cultures were set as specified in Materials and Methods. At the indicated times, samples were labeled with Van-FL and observed under a fluorescence microscope. (A) Filaments were photographed. Arrows indicate fluorescence matching divisome localization. Magnification is the same for all micrographs. (B) Lateral and septal fluorescence was quantified as described in Materials and Methods. Student's *t* test was used to assess the significance of differences. For lateral labeling, *P* < 0.01 for comparisons of 24 h, 48 h, or 72 h with 168 h or 216 h. For septal labeling, *P* < 0.01 for 24 h or 72 h in comparison to 168 h or 216 h and *P* < 0.05 for comparisons of 48 h with 168 h or 216 h. For the ratio lateral/septal (L/S), *P* < 0.01 for comparisons of 24 h, 48 h, or 72 h with 168 h or 216 h, and *P* < 0.05 for 24 h against 72 h (Data Set S1). (C) Fluorescence was recorded (lower panel) along a representative stretch of a 24 h-incubated filament (upper panel) in the area covered by the manually defined white line. (D) In the same filament used in (C), total septal fluorescence and septal width were measured in 28 consecutive septa, and the mean fluorescence and mean width were calculated for septa occupying the 14 odd and the 14 even positions. *P* = 0.00022 and *P* < 0.00001 for comparisons of total septal fluorescence and septal width, respectively, between the two classes of septa. (E) Increase in the absorbance at 750 nm (A_750_) of cultures incubated under the same conditions as those used in (A to D) (data from three independent cultures, denoted by different colors are presented). At, absorbance at the corresponding time; A0, absorbance at the start of culture.

Van-FL is a derivative of the antibiotic vancomycin that binds to the terminal d-Ala-d-Ala of PG precursor lipid II once externalized, either bound or not to the sacculus, but not involved in cross-linking, thus providing a probe for nascent PG synthesis (25). Some previous reports that included Van-FL-staining of *Anabaena* filaments are available (e.g., (32)). Here, we aimed to compare the patterns of PG growth in different growth phases in *Anabaena* and mutants impaired in cell division or elongasome factors. Fig. 1A shows images of *Anabaena* filaments stained with Van-FL. Quantitative determination of the labeling at lateral and septal locations is presented in Fig. 1B Under exponential growth (Fig. 1A, 24 and 48 h; see Fig. 1E), Van-FL staining produced weak, mostly homogenous fluorescence at the cell periphery and conspicuous labeling as bands at the intercellular septa between contiguous cells in the filaments. Additionally, labeling was observed at the midcell, matching the nascent septum in dividing cells. Average septal fluorescence intensity was about 3-fold that of the peripheral fluorescence (Fig. 1B). Notably, fluorescence intensity was higher in alternating older (narrower) septa than in the more recently constructed (wider) septa along the filament (Fig. 1A; a continuous recording in a stretch of a 24-h filament is shown in Fig. 1C, and an estimation of total fluorescence in alternating narrow and wide septa in Fig. 1D). This suggested that septal PG synthesis continues in *Anabaena* after daughter cells start new rounds of cell division, contributing to PG thickening in the intercellular regions. In the phases of nonexponential growth (Fig. 1A, 168 and 216 h), peripheral fluorescence decreased (Fig. 1B), likely reflecting a lower activity of cell growth. Septal labeling along the filament, although also decreased, was still readily detected (Fig. 1A and B), suggesting persistence of septal PG incorporation, and it was more homogeneous than during exponential growth (Fig. 1A), likely reflecting a lower frequency of cell division. This pattern results in a decrease in the average ratio of lateral versus septal labeling in comparison to the phases of exponential growth.

10.1128/mbio.01165-22.1 DATA SET S1 Statistical test of data on Van-FL fluorescence intensity. Download Data Set S1, XLSX file, 0.02 MB. Copyright © 2022 Velázquez-Suárez et al. 2022 Velázquez-Suárez et al. https://creativecommons.org/licenses/by/4.0/ This content is distributed under the terms of the Creative Commons Attribution 4.0 International license.

### Localization of MreB, MreC and MreD in *Anabaena*.

**FIG 2:**
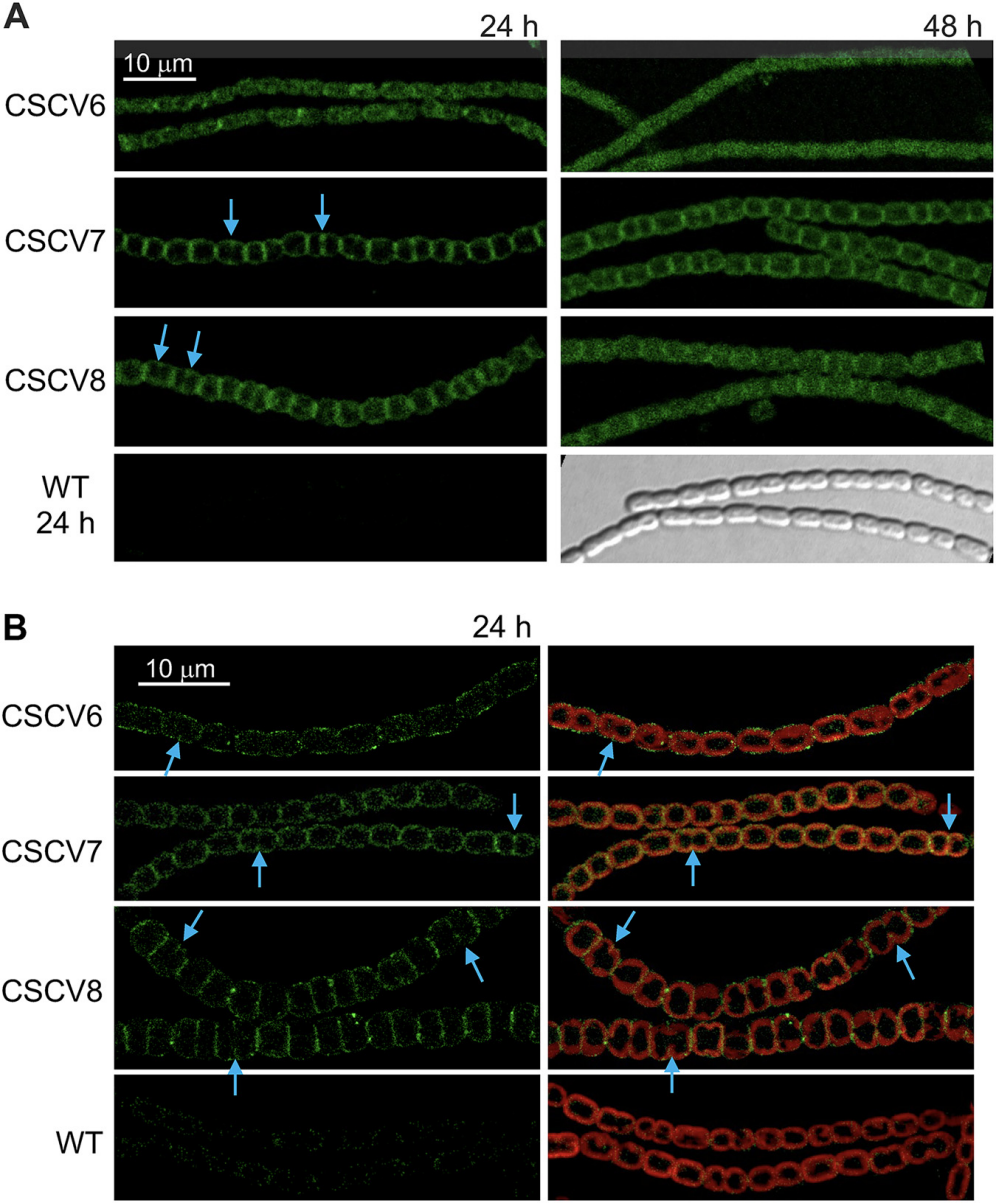
Localization of MreB, MreC and MreD in *Anabaena*. Exponential cultures of strains PCC 7120 (WT) and their derivatives CSCV6 (sfGFP-MreB), CSCV7 (sfGFP-MreC), and CSCV8 (sfGFP-MreD) were set, and, at the indicated times, filaments were visualized by confocal microscopy with a TCS (A) or FLUOVIEW (B) equipment. GFP fluorescence (green), bright-field images, and merged GFP and cyanobacterial autofluorescence (red) are shown. Arrows point to GFP fluorescence matching divisome localization. Magnification is the same for all micrographs in A or B.

The localization of MreB, MreC, and MreD in filaments of *Anabaena* was studied using protein fusions to green fluorescence protein (GFP). For that, we generated strains CSCV6, CSCV7, and CSCV8 that bear the genes *sfgfp-mreB*, *sfgfp-mreC*, or *sfgfp-mreD* (encoding the superfolder GFP fused to MreB, MreC or MreD, respectively) preceded by the native promoter of the *mreBCD* operon, located on an ectopic chromosomal locus (see Materials and Methods; cyanobacterial strains used in this work are listed in Table S1). Besides, these strains carry the intact *mreBCD* operon in its native location. GFP fluorescence was monitored in filaments of CSCV6, CSCV7, and CSCV8. In the phases of faster growth (Fig. 2, 24 h), strain CSCV6 presented weak fluorescence through the cell periphery (see especially high-resolution images in Fig. 2B) and in some discrete patches in the intercellular septal regions (Fig. 2A). Strains CSCV7 and CSCV8 exhibited high fluorescence around the cell periphery. In Fig. 2A, fluorescence appeared most intense in the septal regions between neighboring cells, an effect to which the superposition of two membrane units in these regions could contribute. In addition, in CSCV7 and CSCV8 (and in CSCV6, although less conspicuously due to weakness of signals) midcell fluorescent signals progressing inwards from the cell periphery, likely matching the progressing septa under construction in the dividing cells, could be detected. It is worth noting that some cultures, especially of CSCV8 after 24 h of incubation, presented cells bigger and rounder than the wild-type cells. This could indicate that GFP-MreD might interfere with MreD function. Nonetheless, the specificity of the GFP fluorescence localization in CSCV8 and its consistency with the signals produced by GFP-MreB (in CSCV6) and GFP-MreC (in CSCV7) support that GFP-MreD is reporting the physiological localization of MreD. In the three strains, the peripheral fluorescence became more diffuse after 48 h of incubation, and the septal signal also diminished (Fig. 2A). In summary, during active growth, MreB, MreC, and MreD localize throughout the cell periphery. Notably, they also localize to the septa that are under construction during cell division, remaining localized in the intercellular septal regions after compartmentalization of the resulting daughter cells.

### Influence of MreB, MreC, and MreD on PG growth.

**FIG 3:**
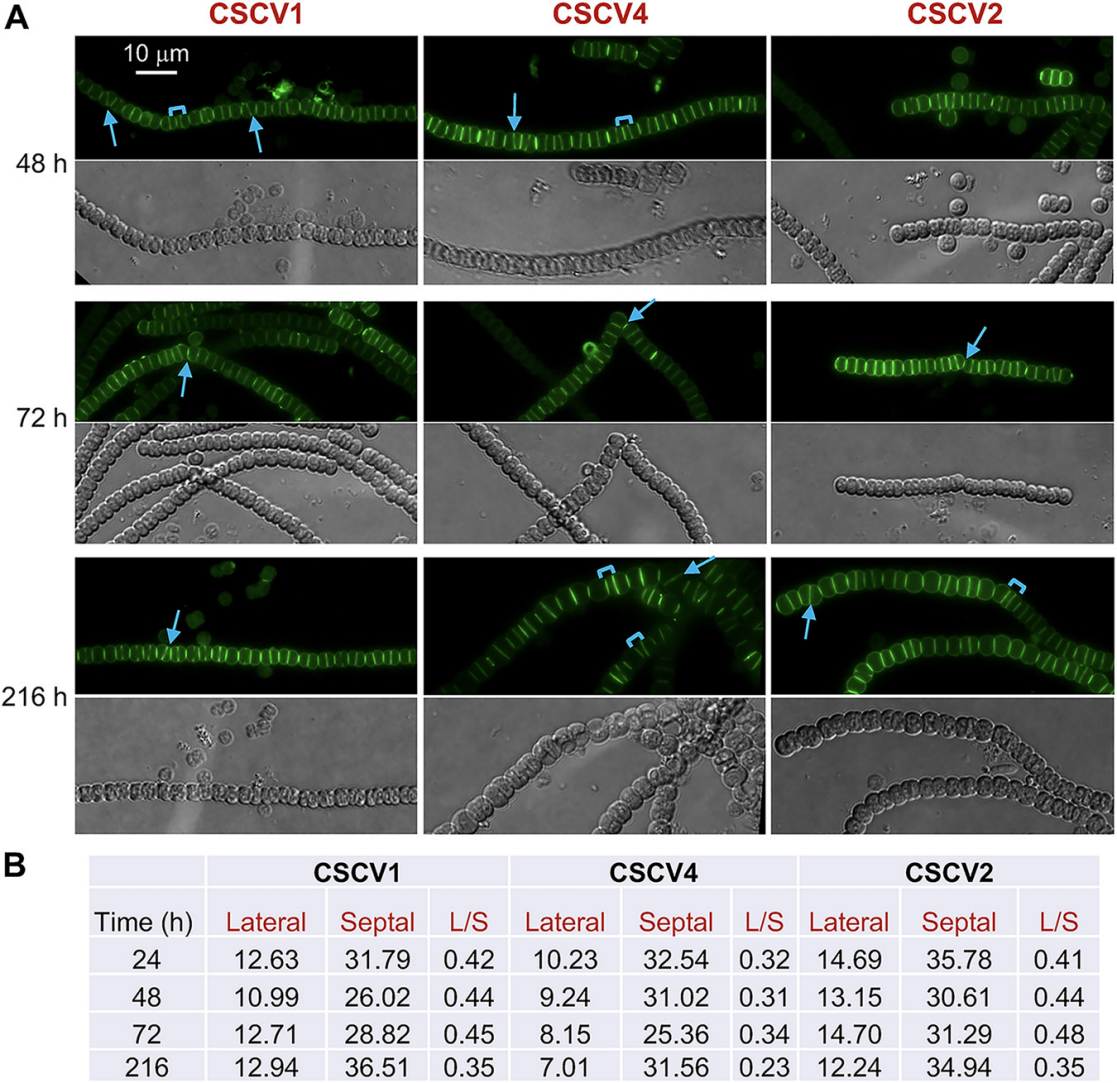
Van-FL staining of *Anabaena mreB*, *mreC*, and *mreD* mutants. Cultures of strains CSCV1 (*mreB*), CSCV4 (*mreC*), and CSCV2 (*mreD*) were set and, at the indicated times, samples were stained with Van-FL and observed under a fluorescence microscope. (A) Filaments were photographed. Van-FL fluorescence (green) and bright-field images are shown. Arrows point to tilted fluorescent bands and brackets to cell compartments of disparate sizes. (B) Lateral and septal fluorescence was quantified and compared to the values obtained for the wild-type (presented in Fig. 1B). For the ratio L/S, *P* values were <0.01 for comparisons of the WT with CSCV1 or CSCV2 at any time, and with CSCV4 at 216 h, and *P* value was <0.05 between the WT and CSCV4 at 48 h (Data Set S1). Magnification is the same for all micrographs.

Van-FL staining was also performed in filaments of strains containing only inactivated versions of *mreB* (CSCV1), *mreC* (CSCV4), or *mreD* (CSCV2), previously generated (30). Despite conspicuous alterations in filament and cell size and morphology (30), strong septal bands of alternating intensity that resemble the septal labeling pattern in the WT, could be observed in filaments of the three mutants (Fig. 3A and Fig. S1). Indeed, the average septal fluorescence intensity in the mutants was somewhat higher than in the wild type (compare Fig. 3B and Fig. 1B). As in the wild type, fluorescence matching the progression of septum formation during cell division could be detected in some cells. However, in the mutants, the septal bands frequently appeared tilted or separating compartments of different sizes. These alterations could explain the observed local deviations in the filament plane and heterogeneity of cell size in the mutants (30) (Fig. 3A and Fig. S1). Thus, MreB, MreC, and MreD are not needed for septal PG synthesis during cell division, but they appear to be needed for the correct positioning of the septal PG growth-plane. On the other hand, peripheral labeling of the mutant round cells was also detected in strains CSCV1, CSCV2, and CSCV4 (Fig. 3 and Fig. S1).

10.1128/mbio.01165-22.2 FIG S1 Van-FL staining of *Anabaena mreB, mreC,* and *mreD* mutants. Filaments of strains CSCV1 (*mreB*), CSCV4 (*mreC*), and CSCV2 (*mreD*) grown in a solid BG11 medium were stained with Van-FL and observed under a fluorescence microscope and photographed. Van-FL fluorescence (green) and bright-field images are shown. Arrows point to tilted fluorescent bands and brackets to cell compartments of disparate sizes. Magnification is the same for all micrographs. Download FIG S1, PDF file, 0.2 MB. Copyright © 2022 Velázquez-Suárez et al. 2022 Velázquez-Suárez et al. https://creativecommons.org/licenses/by/4.0/ This content is distributed under the terms of the Creative Commons Attribution 4.0 International license.

### Localization of FtsZ and ZipN in *mre* mutants.

**FIG 4:**
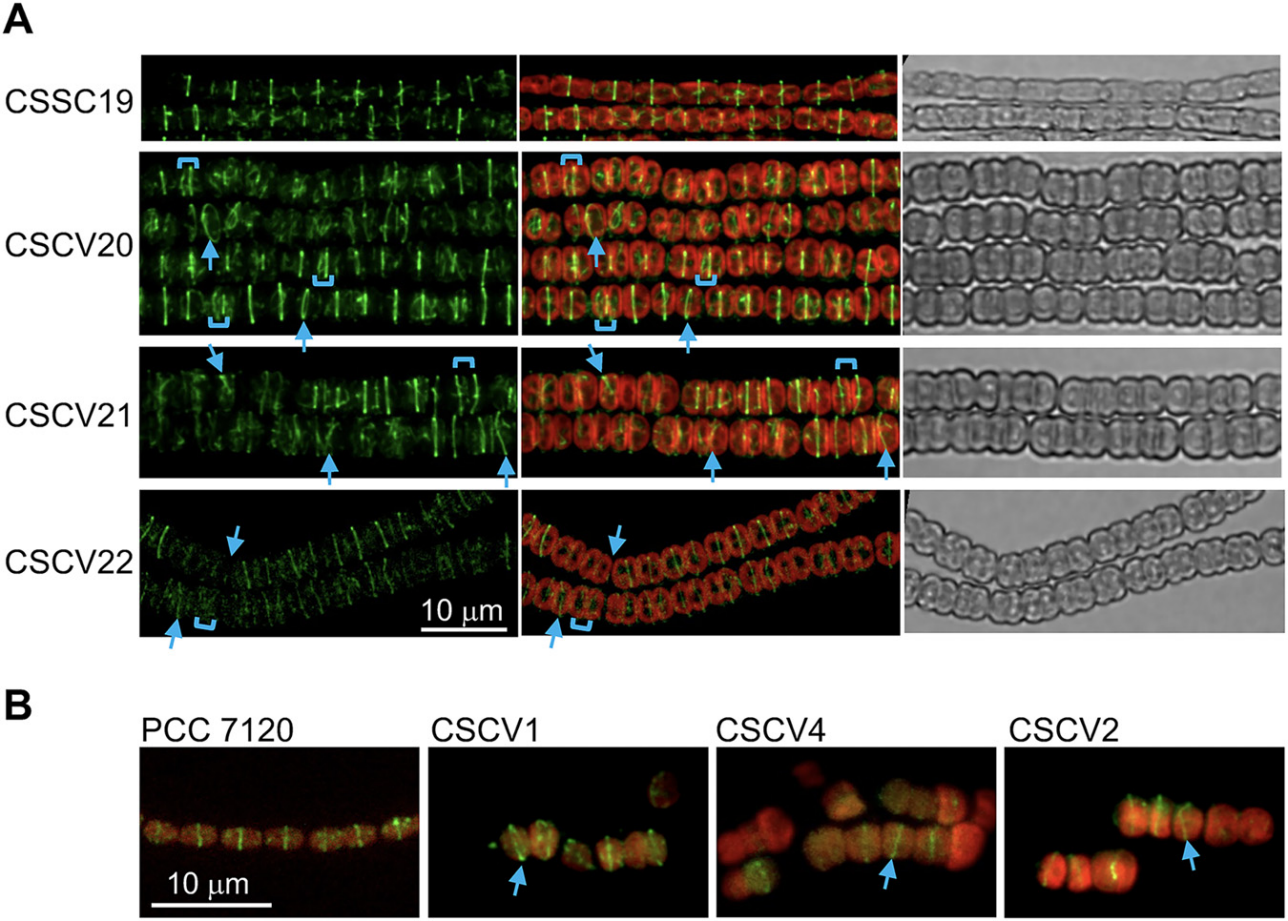
Localization of FtsZ in *Anabaena mreB*, *mreC,* and *mreD* mutants. (A) Cultures of strains CSSC19 (*ftsZ*-*gfp-mut2* in WT background), CSCV20 (*ftsZ*-*gfp-mut2* in *mreB* background), CSCV21 (*ftsZ*-*gfp-mut2* in *mreC* background) and CSCV22 (*ftsZ*-*gfp-mut2* in *mreD* background) were set and, after 48 h, filaments were visualized by confocal microscopy and photographed. GFP fluorescence (green), merged GFP and cyanobacterial autofluorescence (red), and bright-field images are shown. To improve visibility, contrast is higher for the green image of CSCV22. (B) Immunolocalization with antibodies against *Anabaena* FtsZ in the wild-type and strains CSCV1 (*mreB*), CSCV4 (*mreC*), and CSCV2 (*mreD*) incubated for 48 h in liquid BG11 medium (initial cell density, 1 μg chlorophyll/mL). Arrows point to tilted fluorescence bands and brackets to cell compartments of disparate sizes. Magnification is the same for all micrographs in (A or B).

Given the observed instances of misplacement of the medial and septal bands of Van-FL staining in the *mre* mutants, we asked whether the absence of Mre proteins affected the localization of the cell division plane. We then studied the localization of FtsZ-rings and ZipN in the absence of MreB, MreC, or MreD. To study FtsZ, a gene construct, including an *ftsZ-gfp* reporter expressed from the P*_ftsZ_* promoter was transferred to strains CSCV1 (*mreB*), CSCV4 (*mreC*), and CSCV2 (*mreD*), generating strains CSCV20, CSCV21, and CSCV22, respectively. Strain CSSC19 expresses the *ftsZ-gfp* reporter in the WT background (33). As in CSSC19, fluorescent FtsZ-rings were readily detected in strains CSCV20, CSCV21, and CSCV22. However, in the mutant backgrounds some rings were not parallel to those of neighboring cells, and the distance between two consecutive Z-rings was irregular (Fig. 4A). In addition, FtsZ was detected by immunolocalization with antibodies against *Anabaena* FtsZ in the mutants CSCV1, CSCV4, and CSCV2 in comparison to the wild type. Consistent with the results obtained with GFP fusions, some aberrantly located rings could be detected in the mutants (Fig. 4B).

**FIG 5:**
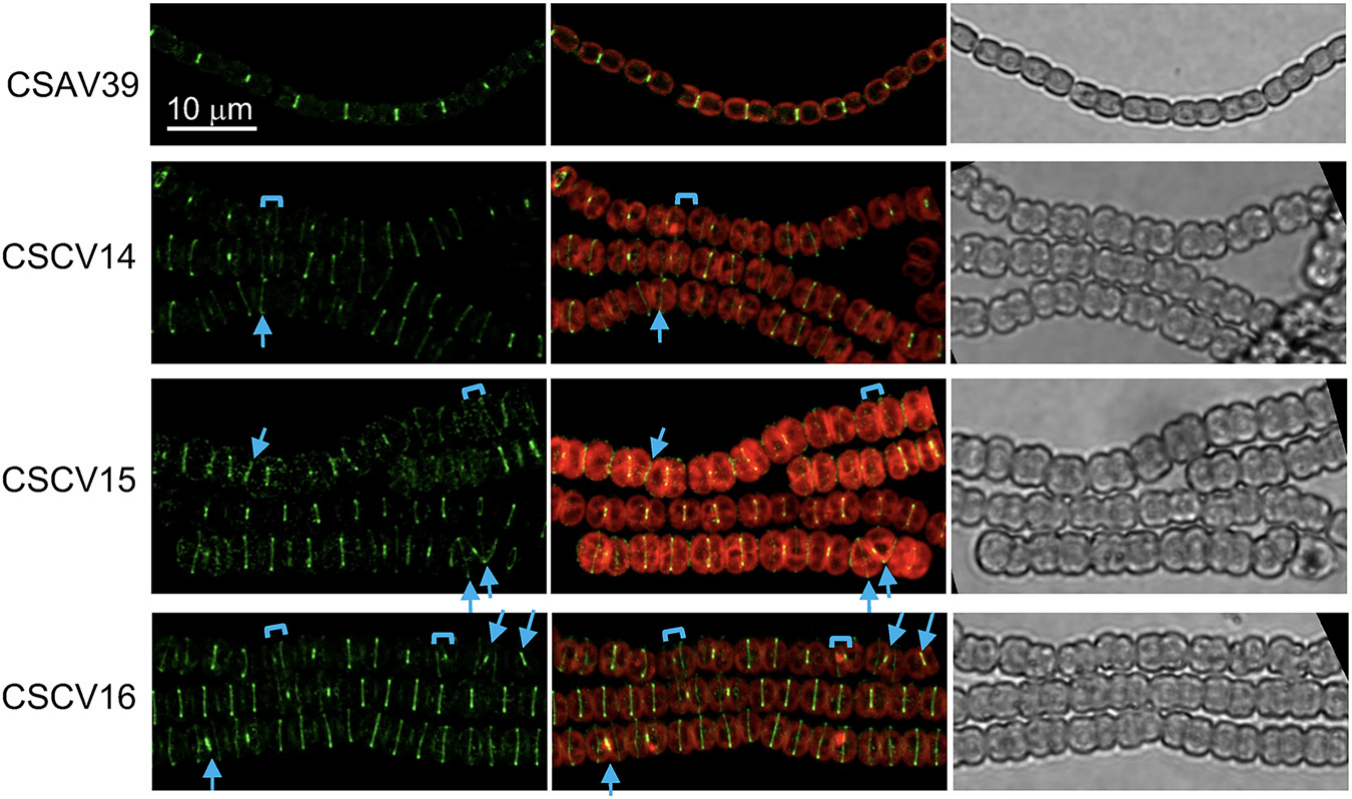
Localization of ZipN in *Anabaena mreB*, *mreC* and *mreD* mutants. Cultures of strains CSAV39 (*sfgfp-zipN* in WT background), CSCV14 (*sfgfp-zipN* in *mreB* background), CSCV15 (*sfgfp-zipN* in *mreC* background) and CSCV16 (*sfgfp-zipN* in *mreD* background) were set and, after 24 h, filaments were visualized by confocal microscopy and photographed. GFP fluorescence (green), merged GFP and cyanobacterial autofluorescence (red), and bright-field images are shown. Arrows point to tilted fluorescence bands and brackets to cell compartments of disparate sizes. Magnification is the same for all micrographs.

To study the localization of ZipN in the absence of MreB, MreC, or MreD, an *sfgfp-zipN* reporter gene expressed from the P*_zipN_* promoter was transferred to strains CSCV1, CSCV4, and CSCV2, generating strains CSCV14, CSCV15 and CSCV16, respectively. In filaments of strain CSAV39 (*sfgfp-zipN* in the wild-type background, (40)), fluorescence was detected in midcell bands and as bands or spots in recently matured septa. In fresh cultures of strains CSCV14, CSCV15, and CSCV16, midcell and septal fluorescent bands or spots could be detected frequently. However, especially in CSCV15, cells exhibiting diffuse fluorescence over the cell periphery were also found (Fig. 5). As with FtsZ-GFP, some neighboring cells exhibited tilted GFP-ZipN bands, and at some places, the distances between consecutive bands in the filament varied.

These results suggest that the misplaced Van-FL-labeled septal bands observed in the *mre* mutants corresponded to places where cell division took place at a plane tilted with regard to that of the previous division or deviated from the midcell position.

### PG growth in *Anabaena* mutants was impaired in FtsZ or ZipN.

**FIG 6:**
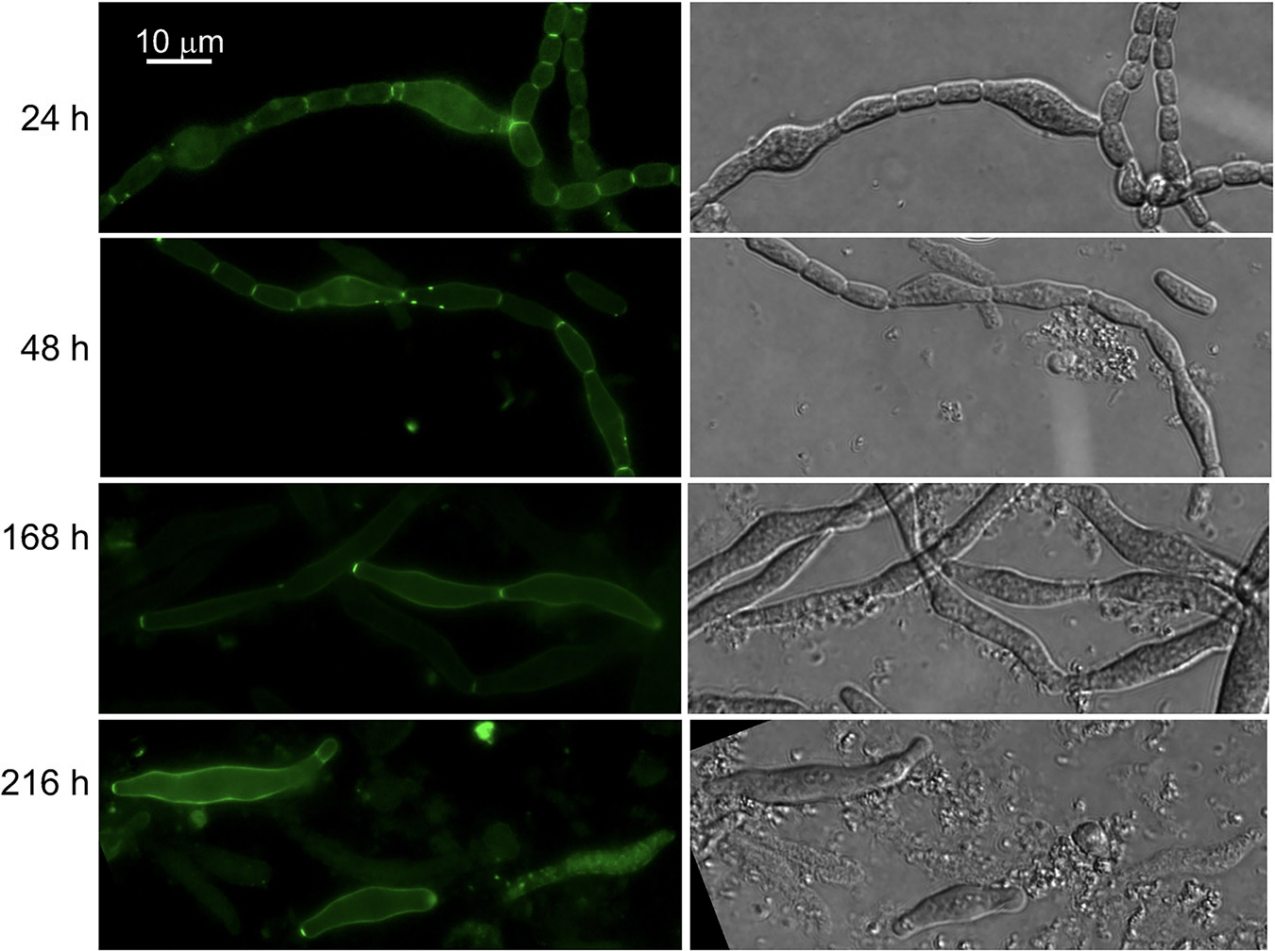
Van-FL staining of strain CSFR18 impaired in FtsZ expression. Filaments of strain CSFR18 (P_ND_-*ftsZ*) (see the text for details) grown in BG11 medium (permissive conditions) were transferred to BG11_0_ + NH_4_^+^ medium (restrictive conditions for *ftsZ* expression) and incubated under culture conditions. At the indicated times, samples were stained with Van-FL, observed under a fluorescence microscope, and photographed. Van-FL fluorescence (green) and bright-field images are shown. Magnification is the same for all micrographs.

**FIG 7:**
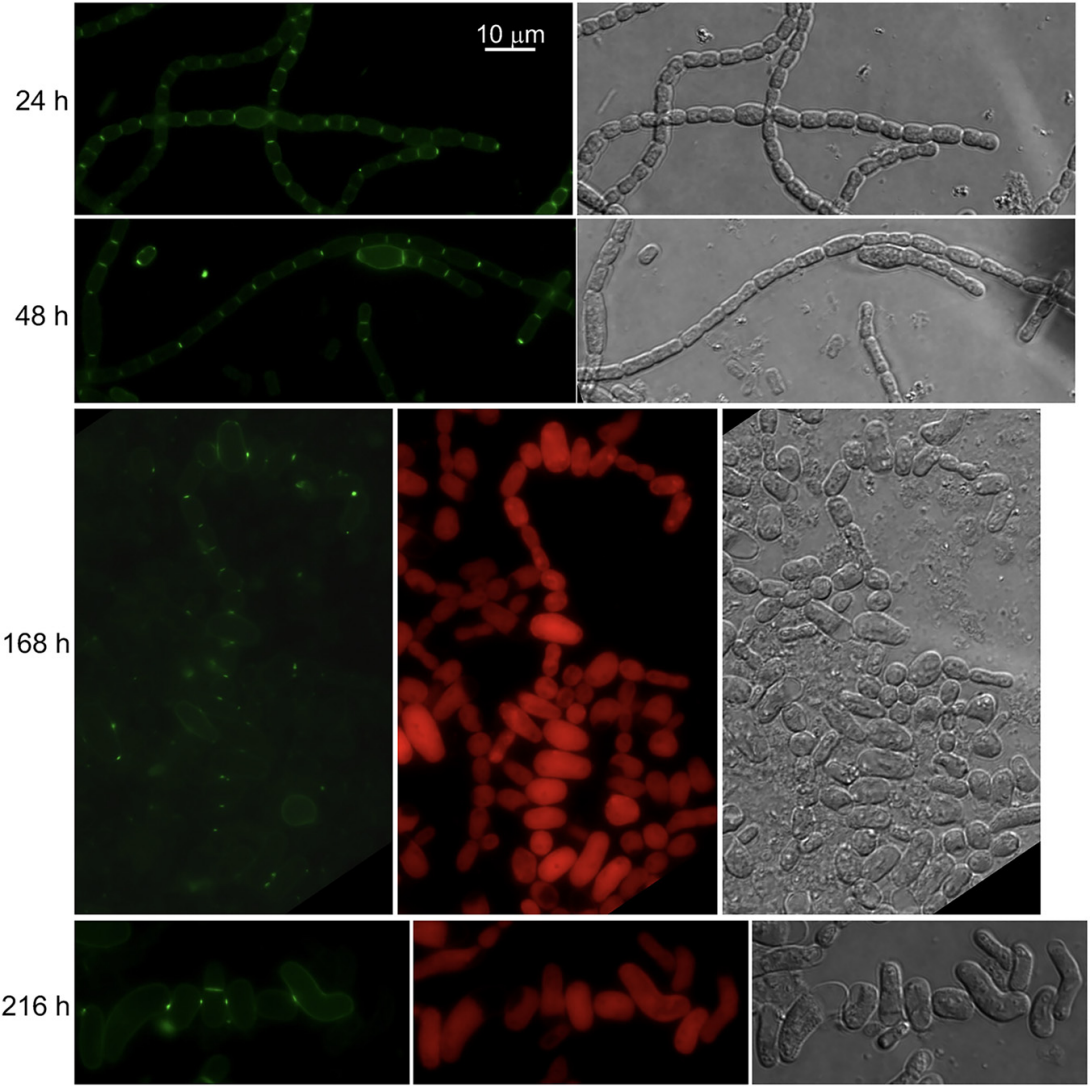
Van-FL staining of strain CSL109 impaired ZipN expression. Filaments of strain CSL109 (P_ND_-*zipN*) (see the text for details) grown in BG11 medium (permissive conditions) were transferred to BG11_0_ + NH_4_^+^ medium (restrictive conditions for *zipN* expression) and incubated under culture conditions. At the indicated times, samples were stained with Van-FL, observed under a fluorescence microscope, and photographed. Van-FL fluorescence (green), cyanobacterial autofluorescence (red), and bright-field images are shown. Magnification is the same for all micrographs.

We have previously described *Anabaena* derivatives that conditionally down-express *ftsZ* (strain CSFR18; (35)) or *zipN* (strain CSL109; (34)) from P_ND_, a synthetic promoter that directs low expression levels in the presence of ammonium. Upon transfer of filaments grown with nitrate (permissive conditions for P_ND_-*ftsZ* or P_ND_-*zipN* expression) to a medium with ammonium (restrictive conditions), strains CSFR18 and CSL109 underwent drastic alterations in cell morphology and extensive cell lysis (see representative images in Fig. 6 and 7, respectively). In the two mutants, morphological alterations preceding cell lysis included cell elongation, bulging of the central cell region, and drastic cell swelling. In CSFR18, long bulged cells could be observed, initially forming filaments, and later as single cells (which likely results from lysis of accompanying cells in the filament). Strain CSL109 presented giant spherical-like cells or ellipsoidal cells in which the axis perpendicular to the filament is longer than that parallel to the filament. These and previous observations (33–35) show that in *Anabaena* impairment of cell division leads to unchecked cell enlargement with restricted elongation producing giant cells with aberrant shapes.

Van-FL staining was performed in strains CSFR18 and CSL109. In filaments incubated with nitrate (permissive conditions), Van-FL staining produced weak peripheral, and strong septal fluorescence signals similar to those observed in the WT (not shown). Upon transfer of strain CSFR18 to medium with ammonium (restrictive conditions for P_ND_-*ftsZ* expression), it showed weak peripheral labeling in the still slightly enlarged cells, and strong dispersed peripheral fluorescence, including occasional discrete spots, in the aberrantly more enlarged cells, which were increasingly abounding (see some examples in Fig. 6). After prolonged incubation under these conditions, most of the remaining cells appeared very deformed and had lost fluorescence, although some very aberrant, mostly isolated cells with conspicuous peripheral fluorescence could also be observed (Fig. 6). In strain CSL109 transferred to ammonium-containing medium (restrictive conditions for P_ND_-*zipN* expression), some cells that began to adopt aberrant engrossed shapes exhibited strong peripheral labeling. However, upon prolonged incubation, most cells showed very weak, progressively fading labeling (Fig. 7).

Regarding midcell staining, no Van-FL labeling was detected in the enlarged cells of CSFR18 or CSL109. Regarding septal labeling, Van-FL bands could be initially detected in the intercellular regions of slightly elongated cells, but they did not increase in width in parallel to the observed increase in cell volume (note enlarged cells connected by tiny septal fluorescent spots in Fig. 6, 48 and 168 h, and in Fig. 7, 168 and 216 h). In CSL109, septal fluorescence was promptly lost, and in CSFR18 many aberrant cells appeared also devoid of septal fluorescence. Thus, the observed septal Van-FL signals appear to correspond to cell division events initiated before restrictive conditions for *ftsZ*, or *zipN* expression were imposed. In summary, in the two strains, the transfer to restrictive conditions leads to the cessation of divisome-associated midcell PG incorporation, but also decreased PG incorporation at the septa and the cell periphery, although a phase of strong peripheral PG incorporation concomitant with severe morphological alterations took place.

### BACTH analysis of protein interactions.

**FIG 8:**
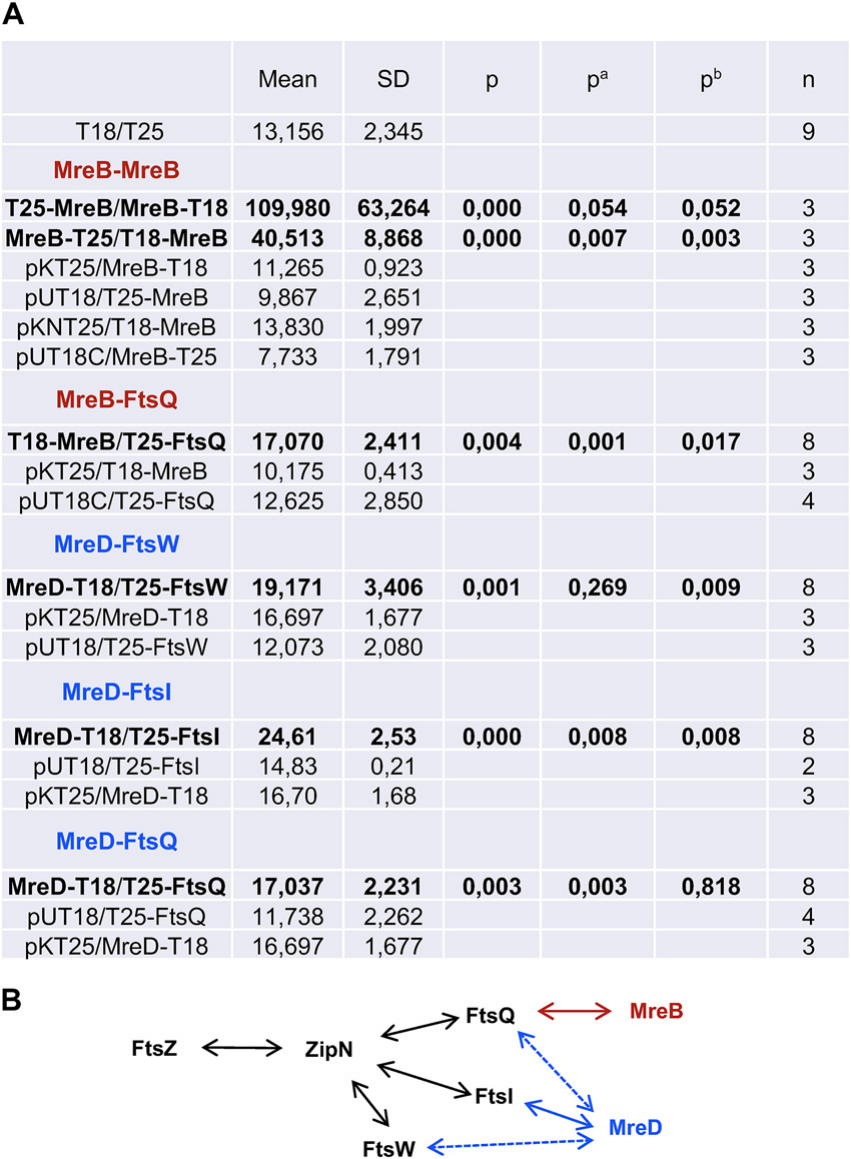
BACTH assay of interactions of Mre and divisome proteins. (A) Interactions of protein pairs produced in E. coli were assayed by measurements of β-galactosidase activity in liquid cultures incubated at 30°C. The topology of each fusion is indicated by the order of components (T18-protein and T25-protein denote the corresponding adenylate cyclase domain fused to the N terminus of the tested protein, whereas protein-T18 and protein-T25 denote fusions to the C terminus). Data are the mean and standard deviation of the indicated number of determinations of the activity (nmol ONP min^−1 ^mg protein^−1^) with the indicated protein fused to T25 (or the empty vectors pKNT25 or pKT25) and the indicated protein fused to T18 (or the empty vectors pUT18C or pUT18). Significance of differences was assessed by Student’s *t* tests: p refers to the test against the strain containing both empty vectors; p^a^ and p^b^ denote tests against the strains expressing each fused protein and the complementary empty vector. (B) Schematic of the positive interactions detected: colored solid lines denote interactions with p, p^a^, and p^b^ <0.02, and dashed lines denote interactions with p and either p^a^ or p^b^ < 0.02. Previously deduced interactions between divisome proteins (33, 34) are depicted in black.

Given the alterations of Z-ring localization in *mreB*, *mreC,* and *mreD* mutants, and the localization of Mre proteins also to division rings in dividing cells, we tested direct interactions between Mre and cell division proteins. We used a bacterial two-hybrid (BACTH) assay with MreB, MreC, MreD, and the divisome proteins FtsZ, ZipN, ZipS (a cyanobacterium-specific cell division factor, (41)), Alr0487 (putative SepF), Alr1706 (putative FtsE), All1757 (putative FtsX), FtsQ, All7666 (putative FtsK), Alr0718 (putative divisome transpeptidase FtsI), and All0154 (putative divisome glycosyl-transferase FtsW). Besides MreB self-interactions, fully significant, although weak, positive interactions were detected with the pairs: T18-MreB/T25-FtsQ and MreD-T18/T25-FtsI (Student’s *t* tests <0.02 for comparisons with all the three controls). In addition, the results obtained suggested interactions between MreD-T18/T25-FtsW; and MreD-T18/T25-FtsQ (Fig. 8A).

## DISCUSSION

In the rod-shaped cells of *Anabaena* sp. strain PCC 7120, Van-FL staining produced weak continuous peripheral fluorescence in the cylindrical part of the cells (Fig. 1; see also reference (32)). This pattern differs from that of weak tilted bands and strong peripheral dots detected in other rod-shaped bacteria such as Bacillus subtilis (25) or C. crescentus (9), which is indicative of a disperse mode of PG incorporation in the cylindrical part of the cell. In B. subtilis, this peripheral PG incorporation is independent of MreB but dependent on Mbl (a MreB homolog), MreC, and MreD (42). Both MreB (8) and Mbl (43) form filaments that rotate around the cell's long axis, and MreC and MreD localize in a similar banded pattern (42). In E. coli, MreB also forms peripheral filaments, whereas MreC and MreD were uniformly localized through the cell periphery (44). In C. crescentus, MreB and MreC form nonoverlapping periplasmic spirals along the perimeter of the cell and, in contrast to predivisional MreB localization at midcell, MreC is not present at the division site (45). Here, we show that in *Anabaena*, MreB, MreC, and MreD localize throughout the cell periphery (Fig. 2). In the presence of combined nitrogen none of the three *mre* genes is essential (30), but mutants lacking any of them form rounded cells or even cells in which the longer axis is perpendicular to the filament. These results indicate an involvement of MreB, MreC, and MreD in cell elongation ((30); Fig. 3). However, the detection of Van-FL peripheral labeling in the round cells of the mutants suggests that some PG incorporation, likely serving maintenance functions, can take place in them. It is worth noting that the pattern of Van-FL staining shown here for *Anabaena*, with weak peripheral and strong septal labeling, is similar to that observed with the fluorescent amino acid HADA that can be incorporated into PG (46), thus precluding that our observations were due to poor access of Van-FL to the *Anabaena* periplasm or Van-FL staining artifacts.

*Anabaena* also showed Van-FL staining in the progressing septum under construction during cell division, and intense bands in the intercellular regions along the filament, even under phases of slow growth (Fig. 1), suggesting that septal growth is maintained during the cell cycle. In addition, the observed alternance of septal Van-FL band intensities (stronger in the older septa and weaker in the more recent ones) is indicative of the persistence of septal PG growth or remodeling after cell division has been completed. During the phases of less active growth, the intensity of fluorescence in septal bands homogenize through the filament and remains strong, also suggesting septal PG growth/remodeling after cell division. This contrasts with the pattern in *Bacillus* in which little polar Van-FL staining is observed in mature cells (25), which is consistent with a narrow period of septal PG growth after which the cell poles remain inert during most of the cell cycle.

In E. coli, preseptal elongation from the midcell is observed only at the initiation of cell division (12), and after completion, the cell poles are inert for PG synthesis (47). In C. crescentus zonal PG synthesis near the midcell takes place between a period of dispersed lateral growth and cell constriction (13). In S. pneumoniae, PG synthesis is halted after completion of cell division and the resulting cell poles become inert (25). In contrast, in the case of C. glutamicum, PG growth directed by the divisome remains active after cell division has completed, so that growth continues at the cell poles leading to a rod-like shape (25). These models differ from the situation in *Anabaena*, in which the persistent and strong septal labeling appears restricted to the intercellular regions of the filament, consistent with the thickening of the PG layer observed in these regions.

In *Anabaena*, our observations with strain CSCV6 (*sfgfp-mreB*) suggest the localization of MreB throughout the cell periphery, including the septal regions as well as in the divisome (Fig. 2). These observations could be compared with two previous reports: one with a *gfp*-*mreBCD* construct overexpressed from a heterologous promoter in a replicative plasmid, which gave conspicuous fluorescent spots at the cell poles (31), and another with *gfp*-*mreB* expressed from P*_mreB_* in a replicative plasmid, which showed filaments dispersed through the cell without any directional preference (48). On the other hand, strains CSCV7 (*sfgfp-mreC*) and CSCV8 (*sfgfp-mreD*) allowed the localization of MreC and MreD, respectively, at midcell matching the growing septum in dividing cells, even at advanced stages of septum formation, and in the intercellular regions between adjacent cells in the filament after septum closure as well as in nondividing cells (Fig. 2). Indeed, in *mreB*, *mreC* and *mreD* mutants, intercellular septa are wider and septal Van-FL labeling signal is stronger than in the wild type, likely contributing to increase filament length and rigidity (compare Fig. 1 and 3; (30)). These results suggest that, although MreB, MreC, and MreD are not required for septal PG synthesis, they participate in septum constriction and intercellular PG maintenance at all stages of the cell cycle.

In contrast to the wild type, in which the new septa localize at midcell in parallel to that of the previous division and perpendicular to the filament long axis, in a *mreB*, *mreC,* or *mreD* mutant septal PG bands are frequently found tilted with regard to those of previous divisions, a disposition never observed in the wild type, or separating compartments with different sizes (Fig. 3). These alterations appear to cause the observed variability of cell size and distortions in the filament plane, even leading to apparent branches, in the mutants (30) (Fig. 3 and Fig. S1). Furthermore, we have shown here that during cell division, whereas in the wild-type the proteins FtsZ and ZipN localized to parallel Z-rings in consecutive cells, in the *mreB*, *mreC*, and *mreD* mutants some tilted and diverted FtsZ (Fig. 4) and ZipN (Fig. 5) rings are observed. Also, especially in the *mreC* mutant, fluorescence from GFP-ZipN appeared somewhat dispersed (Fig. 5). Thus, in the mutants the alterations in the localization of the septal PG incorporation likely originate from misplacement of the Z-ring. Therefore, although MreB, MreC, and MreD are not required for divisome-associated PG growth, they are needed for its correct geometric localization. The altered localization of the division plane might result, at least in part, from the increased cell volume and rounded cell morphology of the *mre* mutants. However, a more direct effect of the Mre proteins localized at the division site is possible, as supported also by our BACTH results suggesting direct interactions involving MreB and MreD and the divisome components FtsQ, FtsW, and FtsI (Fig. 8).

In *Anabaena*, the morphological alterations observed when the initial steps of cell division are inhibited differ from those reported for other bacteria. Hence, in E. coli FtsZ depletion (49) or mutation (50) leads to the formation of long filaments; in B. subtilis, FtsZ depletion leads to cell elongation but does not affect growth rate (25), and in C. crescentus FtsZ depletion leads to cell elongation by a disperse mode of PG synthesis over the sidewalls (13). Thus, in E. coli, B. subtilis, and C. crescentus, lateral PG growth to elongate the cells can be maintained in the absence of cell division. In contrast, *Anabaena* derivatives that conditionally down-express FtsZ or ZipN show strong deformations of the rod-shape morphology normally exhibited by the wild type, or by the mutants under permissive conditions, forming enlarged cells with irregular width (larger in the central region than in the polar regions), more abundant under FtsZ shortage, and giant cells with inverted polarity in the filaments that progress to lysis under ZipN shortage (34, 35) (Fig. 6 and 7). Upon transfer to repressive conditions, Van-FL staining of strains CSFR18 (P_ND_-*ftsZ*) and CSL109 (P_ND_-*zipN*) revealed a phase of strong abnormal PG incorporation through the periphery of aberrant cells, apparently followed by cessation of PG incorporation. Interestingly, it has been previously described that treatment of *Anabaena* with aztreonam (an inhibitor of FtsI) leads, in the short term, to strong peripheral HADA incorporation (46). Moreover, PG-growth in intercellular septa also ceased leading to the presence of minuscule septa, in comparison to the increased cell volume, joining the aberrant enlarged cells. In summary, in *Anabaena*, inhibition of the initial steps of cell division also inhibits the incorporation of PG at mature filament septa that are observed in the wild type and affects the peripheral PG growth preventing sustained cell elongation without widening.

All present and previous results are consistent with a model in which three modes of PG growth operate in *Anabaena*, namely, peripheral, divisional, and intercellular septal. The peripheral mode requires MreB, MreC, and MreD. The divisional and septal PG growth could proceed in the absence of MreB, MreC, and MreD, although these factors have a role in the correct localization of divisional PG growth at midcell and the correct septal PG dimensions. As was the case for many septal proteins of *Anabaena*, we propose that the localization of Mre proteins to division rings serves to place them in the mature intercellular septa, where the proteins remain after cell division has completed. Septal Mre proteins might represent a topological determinant and regulate the extent of peripheral PG growth. Finally, continuous activity of PG growth and remodeling in the mature intercellular septa could contribute to the maintenance of the inserted septal junction arrays that mediate intercellular communication.

## MATERIALS AND METHODS

### Strains and growth conditions.

*Anabaena* sp. Strain PCC 7120 and mutant strains were grown in a BG11 medium (containing NaNO_3_ as a nitrogen source) (51). Cultures were incubated at 30°C with illumination (12 μΕ m^−2^ s^−1^ white light emitted from Osram LED lamps 16.4 W/4000K) in Erlenmeyer flasks with shaking or in plates of medium solidified with 1% Difco agar. For the mutants, media were supplemented with antibiotics: spectinomycin dihydrochloride pentahydrate (Sp) and streptomycin sulfate (Sm) at 5 μg mL^−1^ each in solid medium or 2.5 μg mL^−1^ each in liquid medium (CSCV2, CSCV6, CSCV7, CSCV8, CSFR18, CSSC19, CSL109, CSAV39), with neomycin sulfate (Nm) at 25 μg mL^−1^ in solid medium or 5 μg mL^−1^ in liquid medium (CSCV1, CSCV4), or with Sm, Sp, and Nm (CSCV14, CSCV15, CSCV16, CSCV20, CSCV21, CSCV22). Unless otherwise specified, for the experiments described in this work BG11-grown filaments of the indicated strains were transferred to (at a cell density of 0.5 μg chlorophyll/mL) and incubated in a BG11-based medium lacking NaNO_3_ and supplemented with 4 mM NH_4_Cl and 8 mM TES-NaOH buffer, pH 7.5. It should be noted that, except for experiments with strains CSFR18 and CSL109, similar results were obtained with filaments incubated with nitrate instead of ammonium (unpublished data). The growth of cultures was monitored by measuring the absorbance at 750 nm in aliquots withdrawn at the indicated times. The chlorophyll content of the cultures was determined after extraction with methanol (52). In *Anabaena*, 1 μg chlorophyll corresponds to ca. 3.3 × 10^6^ cells (53).

### Plasmid and strain constructions.

Strains CSCV6, CSCV7, and CSCV8 bear an *sfgfp-mreB*, *sfgfp-mreC,* or *sfgfp-mreD* fusion gene, respectively, expressed from the native *mreBCD* operon promoter (P*_mreB_*), in a heterologous genomic locus, *thrS2*, which is dispensable under normal growth conditions (54). These strains were generated in two steps. In a first step, the construct P*_mreB_*-*sfgfp* was introduced into the coding sequence of *thrS2*, generating strain CSCV5. For that, the sequence of P*_mreB_* was amplified from *Anabaena* genomic DNA with primers all0087-20/all0087-21 (all oligodeoxynucleotide primers used in this work are listed in Table S2), and the *sfgfp* from plasmid pCSAL39 (55) with primers SF-GFP-F2/SF-GFP-R2 (plasmids used in this work are listed in Table S1). Both DNA fragments were joined together by overlapping PCR and introduced into previously cloned *thrS2* sequence in plasmid pMBLT (Canvas), generating plasmid pCSCV9. (To generate a site for introducing the P*_mreB_*-*sfgfp* construct, the plasmid containing *thrS2* was amplified with primers all4723-3/all4723-4, both corresponding to internal *thrS2* sequences and ending with NsiI restriction sites.) The insert of pCSCV9 was transferred to the conjugative vector pRL277 including genes for Sm^R^/Sp^R^ and *sacB*, encoding susceptibility to sucrose for positive selection (56), generating plasmid pCSCV10. Finally, pCSCV10 was transferred to *Anabaena* by conjugation selecting first for resistance against Sm and Sp, and later for sensitivity to sucrose. One clone that bore the P*_mreB_*-*sfgfp* construct inserted on *thrS2* by double crossover (verified by PCR) was selected (strain CSCV5). In a second step, a DNA fragment encoding *sfgfp* lacking the stop codon (amplified with primers SF-GFP-F/SF-GFP-R) was fused by overlapping PCR to fragments encoding *mreB* (amplified with all0087-22/all0087-23), *mreC* (all0086-13/all0086-14) or *mreD* (all0085-8/all0085-9), preceded by a 5-Gly encoding sequence. The resulting fusions were cloned into the conjugative vector pCSV3 including determinants for Sm^R^/Sp^R^ (57), generating plasmids pCSCV11, pCSCV12, and pCSCV13, which were transferred to strain CSCV5. Clones bearing plasmids pCSCV11, pCSCV12, or pCSCV13 inserted by single crossover into *thrS2* were selected and verified by PCR.

10.1128/mbio.01165-22.3 TABLE S1 Cyanobacterial strains and plasmids used in this work. Download Table S1, DOCX file, 0.02 MB. Copyright © 2022 Velázquez-Suárez et al. 2022 Velázquez-Suárez et al. https://creativecommons.org/licenses/by/4.0/ This content is distributed under the terms of the Creative Commons Attribution 4.0 International license.

10.1128/mbio.01165-22.4 TABLE S2 Oligodeoxynucleotide primes used in this work. Download Table S2, DOCX file, 0.02 MB. Copyright © 2022 Velázquez-Suárez et al. 2022 Velázquez-Suárez et al. https://creativecommons.org/licenses/by/4.0/ This content is distributed under the terms of the Creative Commons Attribution 4.0 International license.

Strains CSCV14, CSCV15, and CSCV16 bear the fusion *sfgfp-zipN* expressed from the P*_zipN_* promoter, together with a native copy of P*_zipN_*-*zipN*, in a CSCV1, CSCV4, or CSCV2 background, respectively. To generate them, the pCSV3-based plasmid pCSAV285, which includes the P*_zipN_*-*sfgfp-zipN* construct (40), was transferred to strains CSCV1 and CSCV4 with selection for Sm^R^/Sp^R^. On the other hand, the insert of pCSAV285 was cloned into the KpnI site of pRL424 (58) generating plasmid pCSCV37, which was transferred to strain CSCV2 with selection for Nm^R^. One clone resulting from each conjugation that had inserted the transferred plasmid by a single crossover into the *zipN* locus leaving an intact P*_zipN_*-*zipN* gene (verified by PCR) was selected.

Strains CSCV20, CSCV21, and CSCV22 bear the fusion *ftsZ-gfpmut2* expressed from the P*_ftsZ_* promoter, together with a native copy of P*_ftsZ_*-*ftsZ*, in a CSCV1, CSCV4, or CSCV2 background, respectively. To generate them, the pRL277-based plasmid pCSSC39, which includes the P*_ftsZ_*-*ftsZ-mut2gfp* construct (33) was transferred to strains CSCV1 and CSCV4 with selection for Sm^R^/Sp^R^. On the other hand, the insert of pCSSC39 was cloned into the SacI/XhoI sites of pRL278 (59) generating plasmid pCSCV39, which was transferred to strain CSCV2 with selection for Nm^R^. One clone resulting from each conjugation that had inserted the transferred plasmid by a single crossover into the *ftsZ* locus leaving an intact P*_ftsZ_*-*ftsZ* (verified by PCR) was selected.

### Van-FL staining and quantification.

For Van-FL staining, filaments were suspended in a liquid medium supplemented with 2 μg/mL Vancomycin-FL (Bodipy-FL conjugate, Invitrogen) and incubated for 1 h in the dark with shaking at 30°C. Filaments were washed twice with liquid medium and spotted in agar. Lateral and septal fluorescence was quantified with ImageJ (60) processing of fluorescence images by collecting total fluorescence in manually defined equal square sections (0.41 μm in length) at the periphery and the intercellular septa of each counted cell. For each cell, lateral fluorescence was calculated as the mean of the values of four sections, and septal fluorescence as the mean of two sections, one at each cell pole. Twenty to thirty cells were counted for each strain and condition, and the average values were calculated. ImageJ was used also for recording fluorescence along determined filament areas (Fig. 1C) and for quantification of septal width and total septal fluorescence using manually defined sections including the whole septa (Fig. 1D).

### BACTH analysis.

BACTH assays based on the reconstitution of adenylate cyclase from Bordetella pertussis (61) were performed. Genes were amplified by PCR using *Anabaena* DNA as the template and oligonucleotide pairs: all0087-10/all0087-11 (T25-MreB), all0087-12/all0087-11 (T18-MreB), all0087-12/all0087-13 (MreB-T25, MreB-T18), all0086-17/all0086-18 (T25-MreC), all0086-19/all0086-18 (T18-MreC), all0085-12/all0085-13 (MreD-T25, MreD-T18), all0154-11b/all0154-13 (FtsW-T18), all1616-4/all1616-5 (T25-ZipS, T18-ZipS), all7666-3/all7666-2 (T18-FtsK) and all7666-3/all7666-4 (FtsK-T25, FtsK-T18), alr1706-4/alr1706-2 (FtsE-T18). The resulting PCR products, which were flanked by PstI/EcoRI ends, were cloned in pUT18, pUT18C, pKNT25 or pKT25 digested with the same enzymes, producing fusions to the 5′ or 3′ ends of the genes encoding the adenylate cyclase T18 and T25 fragments. All the resulting plasmids were verified by sequencing. The fusions encoding MreC-T18, MreC-T25, T18-MreD, and T25-MreD could not be cloned. Other fusions to the genes *ftsZ*, *zipN*, *sepF*, *zipS*, *ftsX*, *ftsI*, *ftsQ*, *ftsW*, and *ftsE* were as previously described (33–35). Plasmids were transformed into E. coli XL1-Blue for amplification. Isolated plasmids were cotransformed into strain BTH101 (*cya*-99), and the transformants were plated on a solid LB medium containing selective antibiotics and 1% glucose. Cotransformants were grown in a liquid medium in the presence of IPTG plus antibiotics supplemented with *o*-nitrophenol-β-galactoside. The *o*-nitrophenol produced per mg of protein versus time was represented, and the β-galactosidase activity was deduced from the slope of the linear function.

### Microscopy.

Van-FL fluorescence was visualized with a Leica DM6000B fluorescence microscope and a FITCL5 filter (excitation band-pass, 480/40; emission band-pass, 527/30), and photographed with an ORCA-ER camera (Hamamatsu). For immunolocalization with antibodies against *Anabaena* FtsZ (35), filaments were treated as specified in reference (34) and visualized by fluorescence microscopy as above. GFP fluorescence was monitored with an Olympus TCS SP2 confocal laser-scanning microscope equipped with an HCX PLAN-APO 63 × 1.4 NA oil immersion objective (excitation, 488-nm; collection, 500 to 540 nm for GFP or 630 to 700 nm for cyanobacterial autofluorescence) or with an Olympus FLUOVIEW FV3000 (hyper-resolution) confocal laser-scanning microscope equipped with a UPlanApo 60 × 1.5 NA oil immersion objective (excitation, 488-nm; collection 500 to 540 nm for GFP or excitation, 640 nm; collection, 650 to 750 nm for cyanobacterial autofluorescence).
