# Supplementary material for: The Role of Mre Factors and Cell Division in Peptidoglycan Growth in the Multicellular Cyanobacterium Anabaena
Source: mBio. 2022 Jul 25;13(4):e01165-22. doi: 10.1128/mbio.01165-22 (PMC9426583; doi:10.1128/mbio.01165-22)
Supplement: FIG S1 [file mbio.01165-22-s0002.pdf]

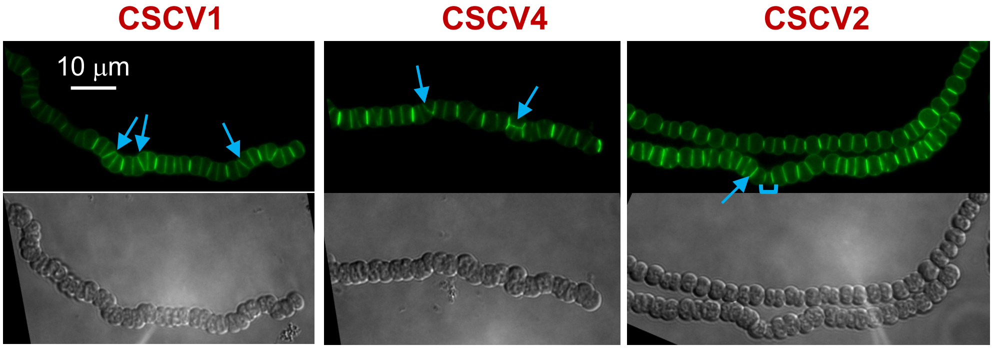


**FIG S1** Van-FL staining of *Anabaena mreB, mreC* and *mreD* mutants. Filaments of strains CSCV1 (*mreB*), CSCV4 (*mreC*) and CSCV2 (*mreD*) grown in solid BG11 medium were stained with Van-FL and observed under a fluorescence microscope and photographed. Van-FL fluorescence (green) and bright-field images are shown. Arrows point to tilted fluorescent bands, and brackets to cell compartments with disparate sizes. Magnification is the same for all micrographs.
