# Supplementary material for: The Role of Mre Factors and Cell Division in Peptidoglycan Growth in the Multicellular Cyanobacterium Anabaena
Source: mBio. 2022 Jul 25;13(4):e01165-22. doi: 10.1128/mbio.01165-22 (PMC9426583; doi:10.1128/mbio.01165-22)
Supplement: TABLE S1 [file mbio.01165-22-s0003.docx]

Table S1. Cyanobacterial strains and plasmids used in this work

| **Strain** | **Genotype** | **Resistance** | **Source** |
| --- | --- | --- | --- |
| *Anabaena* sp. PCC 7120 | WT |  | Pasteur Culture Collection |
| CSCV1 | *mreB*::C.K1 | Nm | 30 |
| CSCV2 | *mreD*::C.S3 | Sm, Sp | 30 |
| CSCV4 | *mreC*::C.K1 | Nm | 30 |
| CSCV5 | *thrS2*::P*_mreB_*-*sfgfp* |  | This study |
| CSCV6 | *thrS2*::P*_mreB_*-*sfgfp*-*mreB* | Sm, Sp | This study |
| CSCV7 | *thrS2*::P*_mreB_*-*sfgfp*-*mreC* | Sm, Sp | This study |
| CSCV8 | *thrS2*::P*_mreB_*-*sfgfp*-*mreD* | Sm, Sp | This study |
| CSSC19 | P*_ftsZ_*-*ftsZ*-*gfpmut2* | Sm, Sp | 33 |
| CSCV20 | P*_ftsZ_*-*ftsZ*-*gfpmut2*, *mreB* | Nm, Sm, Sp | This study |
| CSCV21 | P*_ftsZ_*-*ftsZ*-*gfpmut2*, *mreC* | Nm, Sm, Sp | This study |
| CSCV22 | P*_ftsZ_*-*ftsZ*-*gfpmut2*, *mreD* | Nm, Sm, Sp | This study |
| CSAV39 | P*_zipN_*-*sfgfp*-*zipN* | Sm, Sp | 40 |
| CSCV14 | P*_zipN_*-*sfgfp*-*zipN*, *mreB* | Nm, Sm, Sp | This study |
| CSCV15 | P*_zipN_*-*sfgfp*-*zipN*, *mreC* | Nm, Sm, Sp | This study |
| CSCV16 | P*_zipN_*-*sfgfp*-*zipN*, *mreD* | Nm, Sm, Sp | This study |
| CSFR18 | P_ND_-*ftsZ* | Sm, Sp | 35 |
| CSL109 | P_ND_-*zipN* | Sm, Sp | 34 |

| **Plasmid** | **Description** | **Resistance marker** | **Source** |
| --- | --- | --- | --- |
| pCSAV285 | pCSV3 carrying P*_zipN_*-*sfgfp*-*zipN*) | Sm, Sp | 30 |
| pCSCV10 | pRL277 carrying *thrS2*::P*_mreB_*-*sfgfp* | Sm, Sp | This study |
| pCSCV11 | pCSV3 carrying *thrS2*::P*_mreB_*-*sfgfp*-*mreB* | Sm, Sp | This study |
| pCSCV12 | pCSV3 carrying *thrS2*::P*_mreB_*-*sfgfp*-*mreC* | Sm, Sp | This study |
| pCSCV13 | pCSV3 carrying *thrS2*::P*_mreB_*-*sfgfp*-*mreD* | Sm, Sp | This study |
| pCSCV37 | pRL424 carrying P*_zipN_*-*sfgfp*-*zipN* | Km, Nm | This study |
| pCSCV39 | pRL278 carrying P*_ftsZ_*-*ftsZ*-*mut2gfp* | Km, Nm | This study |
| pCSSC39 | pRL277 carrying P*_ftsZ_*-*ftsZ*-*mut2gfp* | Sm, Sp | 33 |
