# Supplementary material for: The Role of Mre Factors and Cell Division in Peptidoglycan Growth in the Multicellular Cyanobacterium Anabaena
Source: mBio. 2022 Jul 25;13(4):e01165-22. doi: 10.1128/mbio.01165-22 (PMC9426583; doi:10.1128/mbio.01165-22)
Supplement: TABLE S2 [file mbio.01165-22-s0004.docx]

| Name | Sequence (5’-3’) |
| --- | --- |
| all0085-8 | GGTGGAGGTGGAGGTATGCCTTTGTCGCGTTGG |
| all0085-9 | GAGCTGGATCCCTAACTTTCCAACATCTTC*^a^* |
| all0085-12 | TCCGCTGCAGACCTTTGTCGCGTTGGCATC |
| all0085-13 | GCTTGAATTCATACTTTCCAACATCTTCATTC |
| all0086-13 | GGTGGAGGTGGAGGTATGGTTACTGTACGTCGTTG |
| all0086-14 | TTCCGGATCCTCTAGTTGGACTTTTGTTGC |
| all0086-17 | ATAGGTCTGCAGGGTTACTGTACGTCGTTG |
| all0086-18 | AGATTTGAATTCAATCTAGTTGGACTTTTG |
| all0086-19 | AATAGGCTGCAGTGGTTACTGTACGTCGTT |
| all0087-10 | ATATACCTGCAGGGCTTTTTAGGAACTTTCGC |
| all0087-11 | CTGCGAGAATTCCTACATATTTCGAGATCGTC |
| all0087-12 | ACTCAGCTGCAGGGGGCTTTTTAGGAACTTTCG |
| all0087-13 | CGCCGTGAATTCATATTTCGAGATCGTCC |
| all0087-20 | TCTGATGCATCCTTTAGTACATAAG |
| all0087-21 | CTTTGCTCATGCGTCTCTATGCCCCCTATT |
| all0087-22 | GGTGGAGGTGGAGGTGTGGGGCTTTTTAGGAAC |
| all0087-23 | ATTTGGATCCCTACATATTTCGAGATCGTC |
| all0154-11b | GTAATGCTGCAGGAAGCTACGCAGCCTAATTCC |
| all0154-13 | ACTGTTGAATTCTTAAACATCCGCCGACGTTG |
| alr0653-1 | CAACTCCTGCAGGGTTATTAAAACGATCGCTTC |
| alr0653-2 | GATGACGAATTCTAGTGGTTGGTGGTCAGT |
| alr0653-3 | CAAAACTGCAGCTTATTAAAACGATCGCTTC |
| alr0653-4 | GAAAGTGAATTCGTAAAATAATATTTCTGTCGTTGTC |
| all1616-4 | GCTAGGCTGCAGGCGATGGGTCGATGATTC |
| all1616-5 | CTTCTGGAATTCATCATTAGGAATTTTCGT |
| alr1706-2 | ATCCCTGAATTCTTACGATATAATCTACCATC |
| alr1706-4 | TACCAGCTGCAGGGTGCAGTTAAATGG |
| all4723-3 | TAGTATGCATGAGGAAGCTGGCGGTGG |
| all4723-4 | ATAAATGCATTGTAGTTGGGGCTTGC |
| alr5045-7 | TTTAAGCTGCAGGGCTTTACTGCAACCATC |
| alr5045-8 | TATTTAGAATTCATATTATCTGCTTTTTGC |
| alr5045-9 | CTTTAACTGCAGTGGCTTTACTGCAACCAT |
| all7666-2 | AAAGGCGAATTCAGTTCAAATATCATCTTCAC |
| all7666-3 | GAGAAACTGCAGATTAGAAATGTATATTAATAACAAC |
| all7666-4 | GCATGGGAATTCCGAATATCATCTTCACC |
| SF-GFP-F | ACGTAGATCTATGAGCAAAGGAGAAGAACTTTTC |
| SF-GFP-R | ACCTCCACCTCCACCTTTGTAGAGCTCATCCATGCC |
| SF-GFP-F2 | ATAGAGACGCATGAGCAAAGGAGAAGAACT |
| SF-GFP-R2 | ACGTATGCATTTATTTGTAGAGCTCATCCATG |

TABLE S2. Oligodeoxynucleotide primes used in this work

*^a^*The underlined letters indicated a restriction site.
